# Shotgun metagenomics analysis of gut microbiota of three indigenous fish species from the Kizil River, Xinjiang

**DOI:** 10.3389/fmicb.2025.1617701

**Published:** 2025-06-24

**Authors:** Jingru Yang, Zhuang Qiang, Dandan Zhang, Huimin Hao, Jie Wei, Hamid Syeda Maira, Weimin Wang, Zhulan Nie

**Affiliations:** ^1^College of Life Science and Technology, Tarim University, Alar, China; ^2^State Key Laboratory Breeding Base of Protection and Utilization of Biological Resources in Tarim Basin, Xinjiang Production and Construction Crops and Ministry of Science and Technology, Alar, China; ^3^Tarim Rare Fish Research Center, Alar, China; ^4^College of Fisheries, Huazhong Agricultural University, Wuhan, China

**Keywords:** metagenomics, *Schizothorax biddulphi*, *Diptychus maculatus*, *Triplophysa yarkandensis*, gut microbiota

## Abstract

This study investigates the gut microbiota composition and functional adaptations in three indigenous fish species from the Kizil River, Xinjiang: *Schizothorax biddulphi* (SB), *Diptychus maculatus* (DM), and *Triplophysa yarkandensis* (TY), recognizing their ecological significance and the need for conservation insights. Shotgun metagenomics was employed to profile the gut microbiota and functional potential. Taxonomic and functional annotations were analyzed, including identification of dominant taxa, biomarkers (LEfSe), Kyoto Encyclopedia of Genes and Genomes (KEGG) pathways for metabolic functions, and Carbohydrate-Active enZymes (CAZy) database annotations. Environmental parameters (crude oil pollution, nitrogen levels, pathogen presence) were assessed, and dietary shifts during overwintering were characterized. Distinct gut microbiota profiles were identified: Proteobacteria, *Acinetobacter*, and Pseudomonas were dominant overall. Species-specific biomarkers were Micromonospora (DM); Proteobacteria, Firmicutes, Aeromonas, and Bacillus (SB); and Mucoromycota, Vibrio, and Alcanivorax (TY). DM and SB exhibited significantly higher Firmicutes/Bacteroidetes ratios and enhanced nutrient utilization capabilities compared to TY. Key functional pathways included enriched fructose/mannose metabolism (SB) and oxidative phosphorylation (DM). CAZy analysis revealed high CE3 abundance across species, with GT6/GT10 (SB) and PL22 (TY) serving as unique enzymatic biomarkers. Dietary shifts during overwintering occurred: DM and TY transitioned towards herbivory, while SB retained carnivorous tendencies despite increased plant consumption. All species showed reduced immunity, with DM and SB particularly vulnerable to *Acinetobacter*-related infections. Environmental analysis revealed crude oil pollution, elevated nitrogen levels, and contamination with *A. baumannii*. TY demonstrated notable salinity adaptability but heightened sensitivity to pollution. Host phylogeny exerted a strong influence on microbiota composition and metabolic functions. The results demonstrate host-specific microbial adaptation driven by phylogeny. The distinct functional profiles (nutrient utilization, key metabolic pathways like fructose/mannose metabolism and oxidative phosphorylation, CAZy enzymes) reflect ecological niche specialization. The observed dietary shifts and reduced winter immunity, compounded by environmental stressors (crude oil, nitrogen, *A. baumannii*), highlight critical vulnerabilities, especially for DM and SB. TY’s salinity adaptation is counterbalanced by pollution sensitivity. This study provides essential insights for developing targeted conservation strategies and sustainable aquaculture practices for these indigenous species within their natural habitat, emphasizing the need for pollution mitigation.

## 1 Introduction

The gut microbiota plays a critical role in animal growth, forming a symbiotic relationship with hosts through long-term coevolution. Referred to as the “external organ” of the host, it influences nutritional metabolism, immune regulation, and disease prevention (Fan et al., 2023a). While most host-microbiome studies focus on mammals (Sadeghi et al., 2023), fish—comprising over 32,000 species (more than 50% of vertebrate diversity)—offer unique advantages. With a 600-million-year evolutionary history, they exhibit diverse physiological, ecological, and life history traits, making them ideal models for studying host-microbe interactions (Liu et al., 2022; Sadeghi et al., 2023). As a vital protein source for 3 billion people (20% of their intake), fish research is crucial for advancing host-microbiome science (Johny et al., 2022).

The microbial community within the fish intestine forms a complex and specialized ecosystem that supports key biological functions essential to the host, including metabolic processes, growth, development, and immune responses (Riiser et al., 2019). Traditional microbiota studies relying on marker genes (e.g., 16S rRNA) primarily address phylogenetic composition but lack functional insights (Vaishampayan et al., 2010). In contrast, full metagenomics reveals gene abundances and microbial functional potentials, offering comprehensive insights into microflora (Thomas et al., 2012; Xia et al., 2014).

Earlier studies indicate that the gut microbiota of fish is influenced by multifaceted factors, including environmental microbes, diet, species-specific physiology, metabolism, and ecological conditions (e.g., salinity, temperature, dissolved oxygen) (Wong and Rawls, 2012; Talwar et al., 2018; Tyagi et al., 2019; Starr et al., 2024; Kanika et al., 2025). While diet and habitat play roles, host species remains a dominant determinant of microbiome composition and function, with species-specific effects shaping phylogenetic relationships and selecting bacteria adapted to gut environments (Doane et al., 2020; Sadeghi et al., 2023). For instance, herbivorous and carnivorous fish exhibit distinct gut microbial functions (Wu et al., 2025), and the “core gut microbiota” across populations highlights conserved roles in digestion, nutrient absorption, and immunity (Qiang, 2023). However, the specific mechanisms driving microbial variation remain unclear due to the complexity of factor interactions (Sadeghi et al., 2023). Understanding the taxonomic and functional capabilities of fish gut microbiota is crucial for optimizing artificial fish population management. Excessive human exploitation of water resource has degraded the Kizil River ecosystem, severely impacting Xinjiang’s rare native fish habitats and causing rapid wild population declines to endangered levels. Protecting wild fish genetic resources is ecologically significant for sustaining fisheries and human survival.

*Diptychus maculatus* (DM), *Schizothorax biddulphi* (SB), and *Triplophysa yarkandensis* (TY) are endemic fish species in Xinjiang, belonging to Cyprinidae and Cobitidae. DM prefers low-temperature sandy habitats, feeding on benthic animals and algae, and is a national second-class protected species (Qiang, 2023). SB, a plateau-endemic Cyprinidae, inhabits rivers and lakes, feeds on invertebrates and algae, and faces endangerment due to overfishing and habitat loss, being listed as a national second-class protected species (Qiang, 2023). TY, a high-altitude member of Cobitidae, exhibits migratory behavior, with fragmented habitats threatening its survival, and is classified as a regional second-class protected species (Zhao H. et al., 2022). The family *Cyprinidae* is the most diverse family of fishes globally, with 285 genera and 3,023 species (Starr et al., 2024). Fish gut microbiome research has predominantly focused on farmed species (e.g., *Oncorhynchus mykiss*, *Cyprinus carpio*) and model fish due to aquaculture demands, with fewer studies on wild populations, largely hampered by challenges in sampling and obtaining adequate gut contents for analysis (Starr et al., 2024). This gap also applies to Cobitidae species like those in this study. Although certain progress has been made in characterizing fish gut microbiota, of the majority of the studies have focused on model or commercially farmed species, overlooking the regional endemic fish in ecologically unique habitats such as Xinjiang. Furthermore, except for a few studies, the functions of the gut microbiome in fish remain insufficiently explored (Tyagi et al., 2019). Comparative analysis of cross-species functional pathways, such as carbohydrate metabolism or stress resistance, are limited, which constrains their application in conservation and sustainable aquaculture. Metagenomic analysis of fish gut microbial communities is therefore critical to unravel universal metabolic pathways and carbohydrate-active enzyme gene diversity, enabling effective aquaculture management. Understanding the factors shaping microbiota composition and function will also facilitate the development of tools to maintain gut homeostasis and host health. This study aims to provide insights into host-microbiome symbiosis and inform aquaculture practices in Xinjiang.

## 2 Materials and methods

### 2.1 Animals

**Figure 1:**
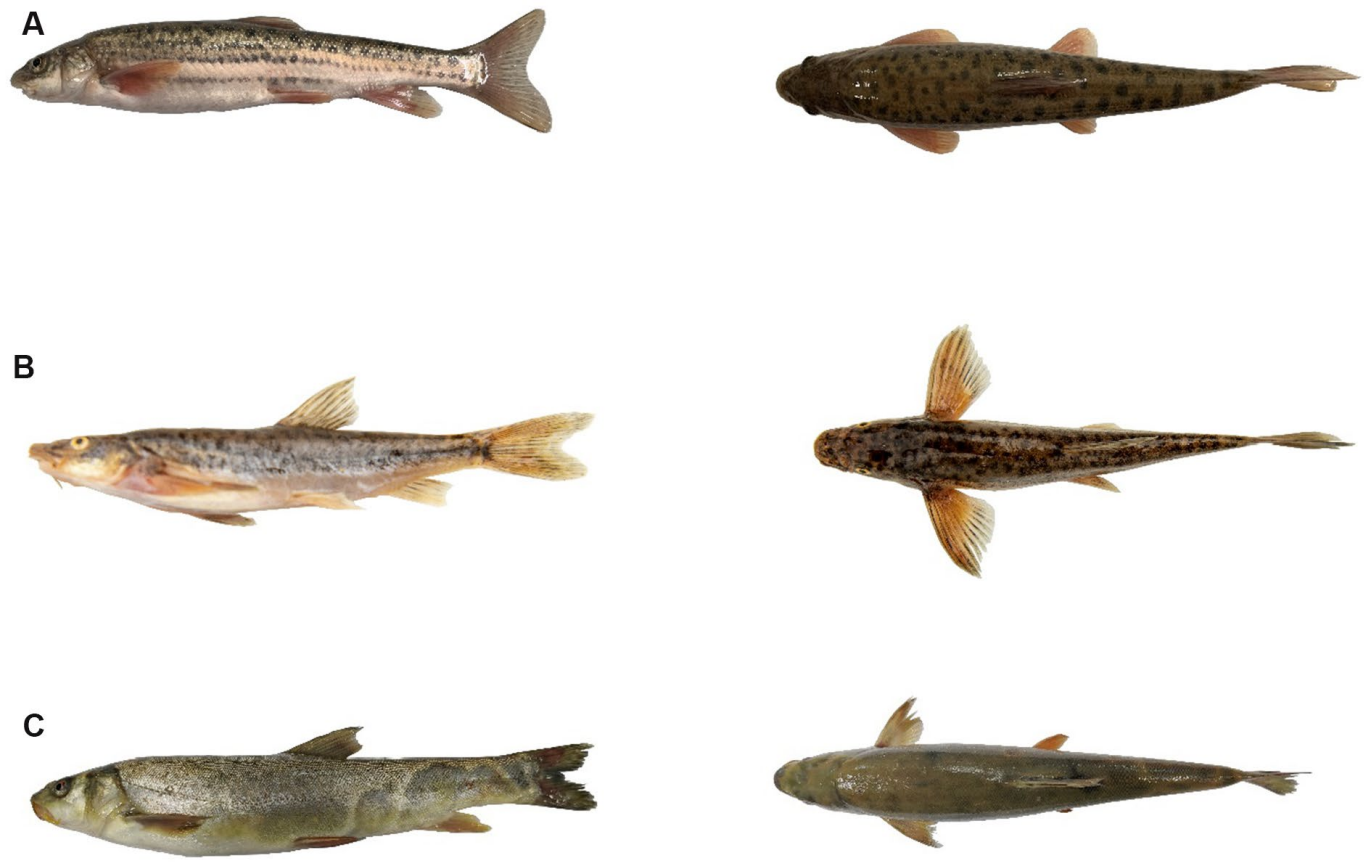
Side view (left) and rear view (right) of three indigenous fish species from Xinjiang. **(A)** *Diptychus maculatus* (DM). **(B)** *Triplophysa yarkandensis* (TY). **(C)** *Schizothorax biddulphi* (SB).

In April 2021, 14 live indigenous wild-type specimens were collected from typical habitats (39°46″–39°50″ N,74°0″–74°15″E) in the upper reaches of the Kizil River (average elevation about 3,300 m), Xinjiang, China. These included six *Diptychus maculatus* (DM), four *Triplophysa yarkandensis* (TY), and four *Schizothorax biddulphi* (SB) (Figure 1). DM has a long body, a conical head, and a lower mouth with a pair of fish whiskers. The mouth is slightly blunt and arcuate, and the lateral line is complete (Hu et al., 2025). The body of TY is fusiform, compressed at the sides, tapering more toward the tail region than the head. The head is short, thick, and compressed dorsoventrally. The body is scaleless and covered with smooth skin (Nie et al., 2013). SB has a long body, a conical head, a pointed snout, and a lower mouth. The scales are small and neatly arranged; there is a bare or scaly chest; the lateral line scales are slightly larger. The lateral line is complete (Wang et al., 2024).

Fish were anesthetized using MS-222 (Guangdong Yufubao Aquatic Technology Co., Ltd., Guangdong, China) as the anesthetic agent. Subsequently, the total length and body weight of each fish were measured with a digital Vernier caliper and an electronic scale, respectively.

For DM, the mean body weight and total length were 212.52 ± 118.42 g and 285.31 ± 58.71 mm, respectively; for TY, they were 12.93 ± 4.75 g and 141.48 ± 39.62 mm; and for SB, the means were 39.45 ± 21.41 g and 171.08 ± 35.69 mm.

### 2.2 Sampling

After euthanasia, all specimens were cut lengthwise in sterile environments to remove the intestine and extract its contents with scissors disinfected in absolute ethyl alcohol, then stored at −80°C in 2 mL Eppendorf tubes. The collected samples were divided into three groups based on species of fish: DM (*n* = 6; DM1, DM2, DM3, DM4, DM5, and DM6), SB (*n* = 4; SB1, SB2, SB3, and SB4), and TY (*n* = 4; TY1, TY2, TY3, and TY4).

### 2.3 Genomic DNA extraction

Genomic DNA was extracted from gut samples using the TGuide S96 Magnetic Bead Fecal Genomic DNA Extraction Kit (BMK-R-0794, Beijing Biomarker Technologies). DNA concentration was quantified using a NanoDrop 2000 spectrophotometer (Thermo Fisher Scientific, United States) and a Qubit^™^ 3.0 Fluorometer (Invitrogen, United States). DNA integrity was evaluated by 1% agarose gel electrophoresis using an EPS600 electrophoresis system (Tanon, China) and an HE-120 electrophoresis tank (Tiangen Biochemical Technology (Beijing) Co., Ltd., China) at 120 V for 30 min, with gel images visualized using a 5200 Multi-image System (Tanon, China).

### 2.4 Metagenome sequencing

Based on the sampling conditions, 14 metagenomic DNA samples collected from 3 indigenous fish species (DM, *n* = 6; SB, *n* = 4; TY, *n* = 4) were processed into qualified libraries and then sequenced via an Illumina NovaSeq 6000 platform using a 150-bp paired-end sequencing strategy (Biomarker Technologies Co., Ltd., Beijing, China). Qualified raw sequencing data were subsequently preprocessed to remove host-derived contamination and assembled *de novo* using MEGAHIT (Li D. et al., 2015) and QUAST (Lin et al., 2017). MetaGeneMark (http://exon.gatech.edu/meta_gmhmmp.cgi, version 3.26, Zhu et al., 2010) was employed to predict genes from the assembled contigs. Using MMseq2 software (https://github.com/soedinglab/mmseqs2, version 11—e1a1c), the non-redundant gene set was built by removing redundancy with a similarity threshold of 95% and a coverage threshold of 90%.

### 2.5 Bioinformatics analysis

#### 2.5.1 Taxonomic composition analysis of species

Species composition and relative abundance were determined based on the sequences in the Nr (non-redundant protein database) that are aligned with non-redundant genes. Python was used to create a bar chart of species at the taxonomic levels of phylum, genus, and species. These charts clearly demonstrate the species composition and relative abundance of different species in each sample. Venn diagrams were created using the VennDiagram package in R (version 3.1.1) to depict the shared and unique features among experimental groups.

#### 2.5.2 Diversity analysis of alpha and beta

Alpha diversity, encompassing within-sample species richness (ACE, Chao1) and evenness (Shannon, Simpson, Pielou’s), was assessed using Good’s Coverage for completeness. Boxplots were generated in R (v3.1.1). Bray–Curtis-based PCoA plots were constructed with Python 2. Unweighted pair-group method with arithmetic mean (UPGMA) clustering of Bray–Curtis distances visualized inter-sample relationships, highlighting compositional differences among samples.

#### 2.5.3 Functional gene composition and abundance analysis

Functional annotation of non-redundant genes was analyzed by BLAST comparison of their protein sequences included in the KEGG database using Diamond (v0.9.29), with a screening threshold *E* value of 1 × 10^−5^. The most similar sequences found in the KEGG database were used to assign annotation information to the corresponding genes in the sequenced metagenomic dataset. Functional annotation was statistically analyzed for the annotation situations of each sample, including the number of pathways annotated. hmmer (v 3.0) software was used to compare the protein sequences of non-redundant genes with the hidden Markov models of each family in the CAZy database, using default comparison parameters. The screening threshold is for alignment >80aa, use *E*-value <1 × 10^−5^; otherwise, use *E*-value <1 × 10^−3^. The covered fraction of HMM >0.3 was required. All identified families that meet the filtering threshold identify the carbohydrate-active enzymes in the genome. In the CAZy function annotation, the number of classes and families annotated to each sample was calculated and analyzed statistically.

#### 2.5.4 Analysis of intergroup differences in species and functional composition

Line discriminant analysis (LDA) effect size (LEfSe python) was applied to identify biomarkers with statistical differences among different groups. This method initially applied the non-parametric Kruskal–Wallis rank sum test to detect species with significant differences in abundance between groups. This was followed by the use of the Wilcoxon rank sum test to compare the differences between subgroups. Finally, linear discriminant analysis (LDA) was adopted to conduct dimensionality reduction analysis on the data and evaluate the influence of species with significant differences. A Log10 (LDA score) >4 was considered a significant abundance difference of the species between the two groups. When the *p*-value <0.05, it indicated that the difference in data was statistically significant.

### 2.6 Statistical analysis

Statistical differences among multiple groups were calculated using a Kruskal–Wallis test. For *p*-values <0.05, significant differences were calculated by the Wilcoxon rank-sum test. PERMANOVA and ANOSIM statistical tests were used to analyze the explanatory power of different grouping factors on sample variation, with permutation testing employed to assess their statistical significance. Both methods yielded R values approaching 1, indicating that between-group differences far exceeded within-group differences, and *p*-values were <0.05, demonstrating high confidence in the test results.

## 3 Results

### 3.1 Analysis of sequencing results

Sequencing and assembly data for the gut microbiota of three Xinjiang native fish species are shown in Tables 1, 2. Deep sequencing yielded 159.87 of Gbp raw data, which was filtered to 123.05 Gbp of clean data (range: 7.63–20.40 Gbp). Total reads amounted to 418.34 M (26.00–70.88 M per sample). Q20/Q30 was ≥96%/90%, and the GC content was 36.37–40.53% (Table 1). Assembly generated 6,970,549 contigs (299,168–600,988 per sample), with a total length of 564.67–749.57 Mbp and the largest contig measuring 16,863–62,921 bp. N50 ranged from 1,476 to 3,901 bp, and the mapping rate was between 98.61 and 99.45% (Table 2). High-quality sequencing and assembly support downstream analyses.

**Table 1:** Sample sequencing information.

Sample ID	Raw data base (bp)	Clean data base (bp)	Number of reads	GC (%)	Q20 (%)	Q30 (%)
DM1	10,540,349,134	7,975,198,855	26,958,907	37.47	97.08	92.37
DM2	10,020,633,900	7,670,842,700	26,001,379	37.33	97.10	92.45
DM3	10,033,286,176	7,667,530,238	26,045,403	37.58	97.43	93.11
DM4	10,027,937,584	7,627,004,388	25,991,367	37.27	96.79	92.08
DM5	10,006,730,518	7,710,728,530	26,084,335	37.32	96.86	91.99
DM6	24,489,331,994	20,398,568,703	70,883,419	40.53	96.22	90.84
SB1	10,848,262,616	8,142,429,487	27,417,700	36.75	97.55	93.87
SB2	10,798,433,954	8,082,986,006	27,323,947	36.37	97.15	92.51
SB3	10,274,964,068	7,761,811,646	26,204,932	36.74	97.43	93.84
SB4	11,669,989,702	8,741,231,630	29,538,936	36.87	97.18	93.33
TY1	10,079,696,576	7,681,376,755	26,224,024	38.54	97.60	93.28
TY2	10,236,924,390	7,945,766,015	26,835,880	38.40	97.59	93.16
TY3	10,725,647,636	7,923,311,537	26,697,311	38.12	97.64	93.22
TY4	10,117,423,530	7,721,442,643	26,132,418	37.88	97.75	93.80

DM, *Diptychus maculatus*; SB, *Schizothorax biddulphi*; TY, *Triplophysa yarkandensis*. Raw data base: the amount of raw data. Clean data base: the amount of data after quality control. Number of reads: the number of reads for the final valid data. GC is the percentage of GC content. Q20 and Q30 refer to the percentage of total bases in clean reads for bases with mass values greater than or equal to 20 and 30, respectively.

**Table 2:** Statistical table of assembly results.

Sample ID	Contig numbers	Total length (bp)	Largest length (bp)	N50 (bp)	GC (%)	Mapped (%)
DM1	542,147	663,647,397	19,067	1,802	36.81	99.16
DM2	576,213	656,313,157	17,411	1,612	36.80	99.07
DM3	586,992	655,410,243	21,887	1,559	36.86	99.13
DM4	600,988	648,912,080	16,911	1,476	36.79	98.74
DM5	560,055	652,035,236	21,724	1,666	36.79	98.98
DM6	541,951	749,569,994	31,721	2,342	37.43	99.44
SB1	572,327	659,045,269	21,904	1,660	36.37	99.17
SB2	585,496	656,769,512	16,863	1,596	36.30	99.10
SB3	578,067	646,948,786	62,921	1,585	36.40	98.61
SB4	570,251	668,883,798	24,264	1,724	36.37	99.06
TY1	346,293	567,719,182	30,601	2,884	38.14	98.85
TY2	299,638	564,673,231	46,385	3,716	38.07	99.43
TY3	299,168	569,806,024	54,071	3,901	38.03	99.45
TY4	310,963	565,928,600	34,725	3,499	38.05	99.15

DM, *Diptychus maculatus*; SB, *Schizothorax biddulphi*; TY, *Triplophysa yarkandensis*. Contig numbers: the number of contigs after assembly. Total length: the sum of the bases of all contigs. Largest length: the longest contig base number. N50 is defined as the length of the contig at which the cumulative length first reaches or exceeds half of the total length of all contigs. (Contigs are arranged in descending order based on their lengths, and the cumulative length is calculated progressively.) Mapped: the ratio of sequencing reads to assembled contigs.

### 3.2 Analysis of species diversity

#### 3.2.1 Community structures of the Xinjiang indigenous fish gut microbiota

**Figure 2:**
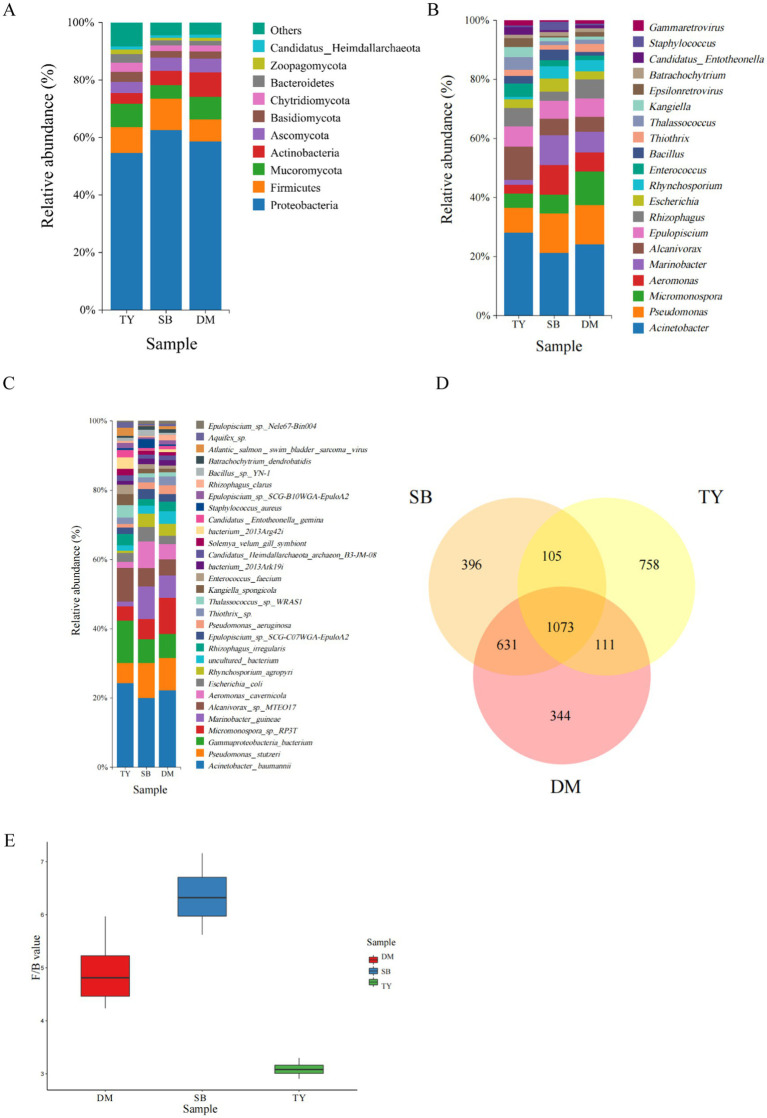
Gut microbiota of three Kizil River indigenous fish species (DM, *Diptychus maculatus*; SB, *Schizothorax biddulphi*; TY, *Triplophysa yarkandensis*). **(A)** Phylum-level relative abundance bar plot (top 10 taxa). **(B)** Genus-level relative abundance bar plot (top 20 taxa). **(C)** Species-level relative abundance bar plot (top 30 taxa). **(D)** Venn diagram of species-level gut microbiome shared/unique taxa. **(E)** Firmicutes-to-Bacteroidetes ratio comparison.

The gut microbial community composition of three Xinjiang native fish species is shown in Figure 2. At the phylum level (Figure 2A), Proteobacteria dominated in all groups: TY (44.58%), SB (55.74%), and DM (50.86%), followed by Firmicutes, Mucoromycota, Ascomycota, and Actinobacteria (specific abundances listed). The Firmicutes-to-Bacteroidetes ratio (Figure 2E) was comparable in DM and SB (*p* > 0.05), both significantly higher than TY (*p* < 0.05).

At the genus level (Figure 2B), Acinetobacter was the dominant genus across all species: TY (12.35%), SB (13.29%), DM (15.01%). TY was further characterized by Alcanivorax and Pseudomonas; SB by Pseudomonas, Aeromonas, and Marinobacter; DM by Pseudomonas and Micromonospora.

At the species level (Figure 2C), *Acinetobacter baumannii* was the most abundant in all groups: TY (12.31%), SB (13.28%), DM (14.99%). TY additionally featured *Gammaproteobacteria bacterium* and *Alcanivorax* sp. *MTEO17*; SB included *Pseudomonas stutzeri*, *Marinobacter guineae*, and *Aeromonas cavernicola*; DM had *Micromonospora* sp. *RP3T* and *Pseudomonas stutzeri*.

A species-level Venn diagram (Figure 2D) revealed that DM and SB shared the most taxa (1,704 species), while TY harbored the highest number of unique microorganisms (758), compared to 344 (DM) and 396 (SB).

#### 3.2.2 Analysis of alpha and beta diversity of species

**Figure 3:**
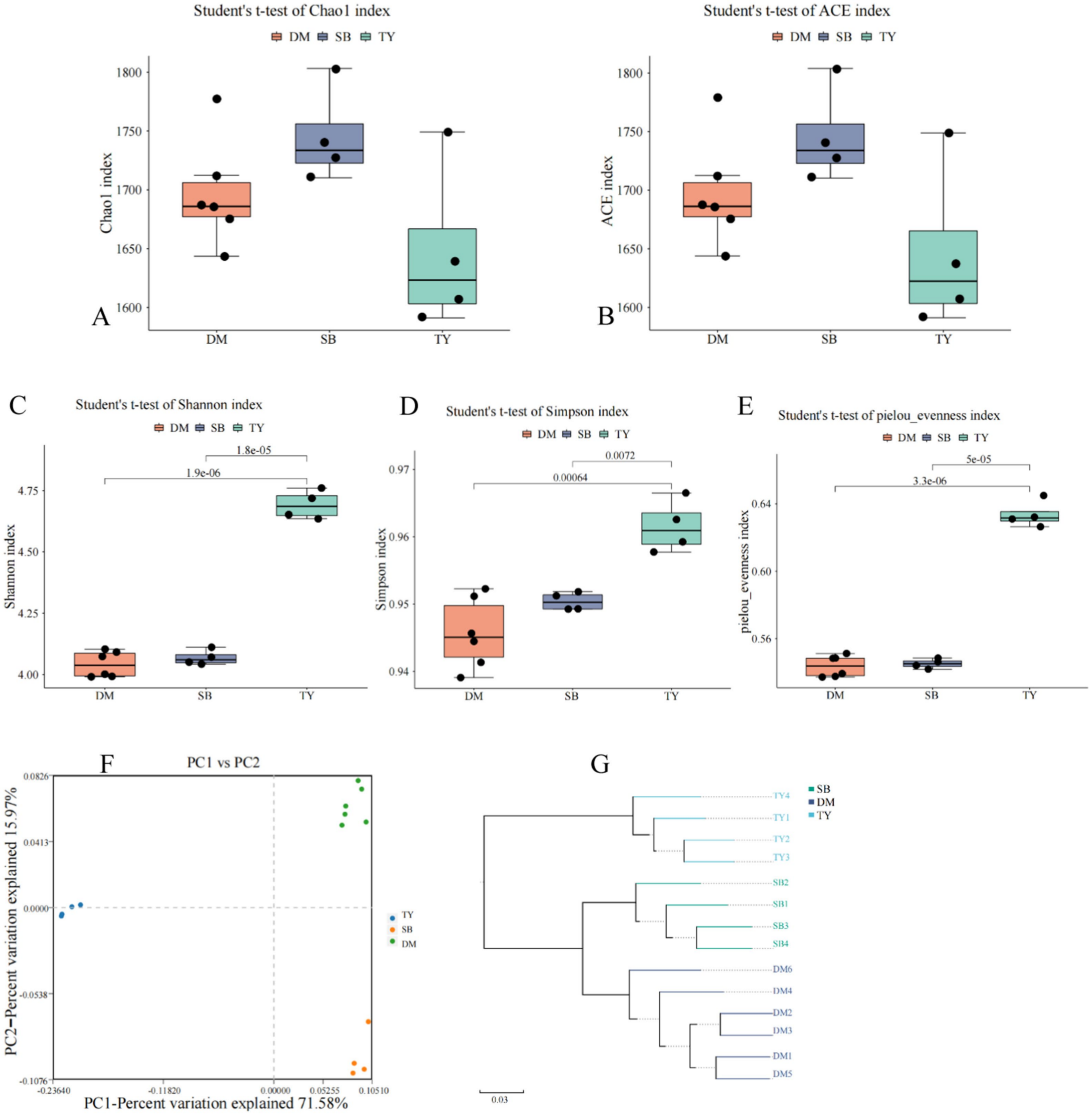
Alpha **(A–E)** and beta **(F,G)** diversity of gut microflora in three Kizil River indigenous fish species. **(A)** Chao1 index. **(B)** ACE index. **(C)** Shannon index. **(D)** Simpson index. **(E)** Pielou evenness index. **(F)** Species-level PCoA via Bray–Curtis (dots = samples, colors = groups; axes: principal components with percentage contributions). **(G)** UPGMA clustering based on species abundance (colors = groups; branch length = compositional similarity). DM, *Diptychus maculatus*; SB, *Schizothorax biddulphi*; TY, *Triplophysa yarkandensis*.

Alpha diversity analysis showed no significant differences in Chao1 and ACE indices among TY, DM, and SB groups (Figures 3A,B, *p* > 0.05), indicating comparable gut microbiota richness. TY exhibited significantly higher Shannon, Simpson, and Pielou’s evenness indices than DM and SB (*p* < 0.05), with no differences between DM and SB (Figures 3C–E).

PCoA revealed distinct spatial separation of DM, SB, and TY samples (Figure 3F), indicating significant differences in species composition. Within groups, samples clustered closely, with DM and TY showing tighter aggregation than slightly dispersed SB samples. Axis 1 and 2 contributed 71.58 and 15.97% of variance, respectively.

UPGMA clustering (Figure 3G) showed intra-group similarity in microbial structures, with DM and SB exhibiting higher functional similarity based on branch length.

#### 3.2.3 Screening of differential microorganisms

**Figure 4:**
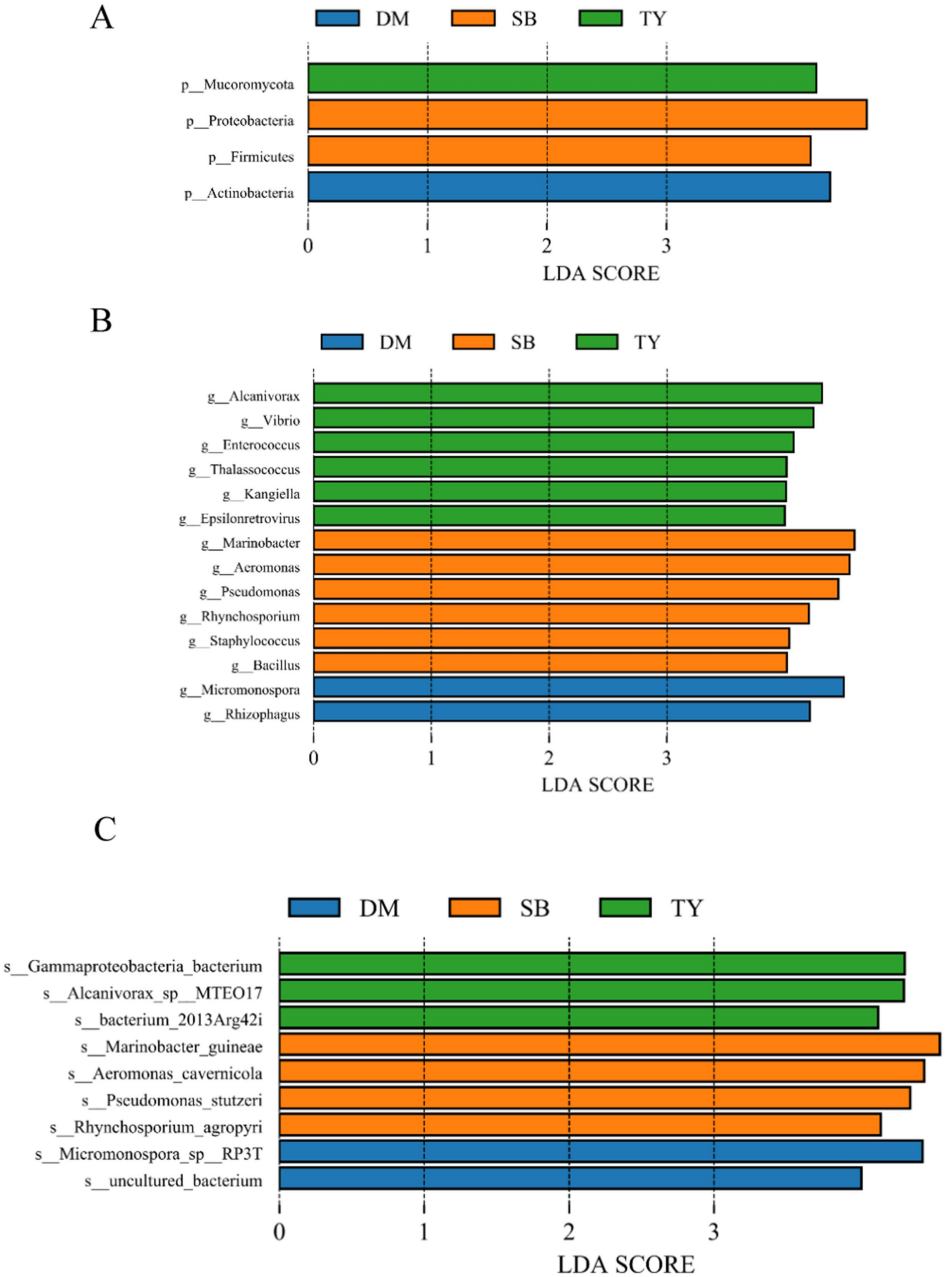
LEfSe gut microbiome analysis (LDA ≥4) of three Kizil River indigenous fish species (DM, *Diptychus maculatus*; SB, *Schizothorax biddulphi*; TY, *Triplophysa yarkandensis*). **(A)** Phylum-level LDA value histogram. **(B)** Genus-level LDA value histogram. **(C)** Species-level LDA value histogram.

LEfSe analysis identified significantly different species biomarkers among TY, DM, and SB groups (Figures 3A,B). At the phylum level (Figure 4A), Actinobacteria differed in DM; Proteobacteria and Firmicutes in SB; and Mucoromycota in TY.

At the genus level (Figure 4B), DM showed Micromonospora and Rhizophagus; SB had Marinobacter, Aeromonas, Pseudomonas, Rhynchosporium, Staphylococcus, and Bacillus; and TY exhibited higher levels of Alcanivorax, Vibrio, Enterococcus, Thalassococcus, Kangiella, and Epsilonretrovirus.

At the species level (Figure 4C), DM had an uncultured bacterium and *Micromonospora* sp. *RP3T*; SB included *Rhynchosporium agropyri*, *Pseudomonas stutzeri*, *Aeromonas cavernicola*, and *Marinobacter guineae*; and TY showed *bacterium 2013Arg42i*, *Alcanivorax* sp. *MTEO17*, and *Gammaproteobacteria bacterium*.

### 3.3 Analysis of functional diversity

#### 3.3.1 Abundance analysis of KEGG functional genes

**Figure 5:**
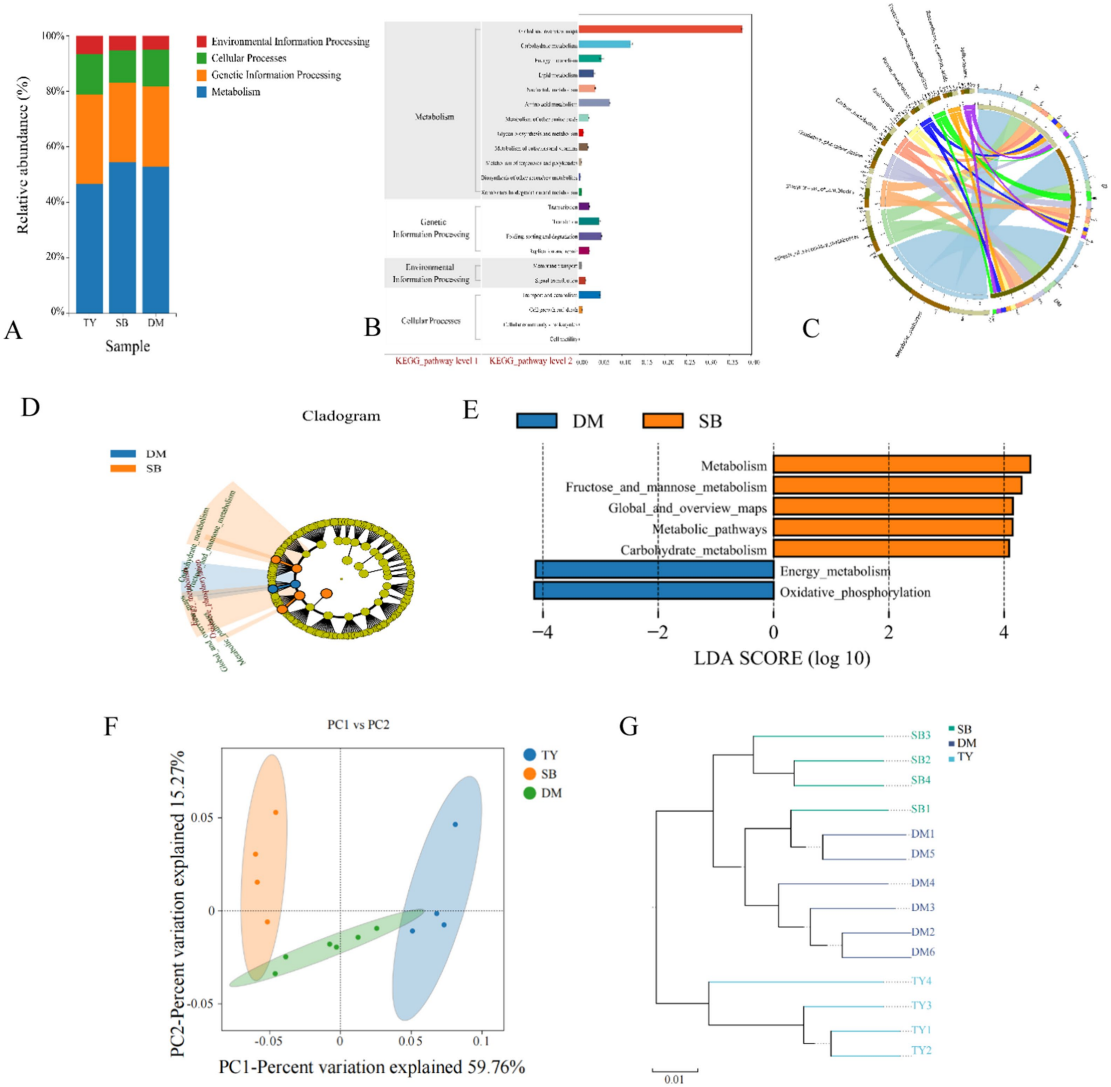
KEGG functional diversity analysis diagram. **(A)** KEGG pathway Level 1 bar chart. X-axis: group names (DM, SB, TY); Y-axis: proportion of Level 1 functional categories per group. **(B)** Statistical chart of KEGG metabolic pathway Level 2 functional genes. X-axis: relative content of functional genes; Y-axis: KEGG Level 2 functional classification. **(C)** KEGG pathway Level 3 circle diagram. One side: sample info; the other: functions (different colors). Ribbons indicate function presence in a sample; width relates to relative abundance (thicker = richer content). **(D)** KEGG pathway LEfSe analysis evolutionary branch diagram. Circles from inside out represent different taxonomic levels. Small circle diameter ∝ relative abundance. Non-significantly different pathways: yellow; others colored by the group with the highest abundance. Different colors = different groups; nodes indicate key metabolic pathways in the group. **(E)** KEGG pathway LEfSe analysis LDA value distribution histogram. **(F)** PCoA of KEGG bray_curtis functional gene abundance. Dots: samples (colors = groups). X/Y-axis: principal characteristic values (percentage influence). **(G)** UPGMA clustering of KEGG bray_curtis functional genes. Colors distinguish groups; branch length: species composition similarity. DM, *Diptychus maculatus*; SB, *Schizothorax biddulphi*; TY, *Triplophysa yarkandensis*.

KEGG pathway annotations were analyzed hierarchically (Figure 5). At Level 1 (Figure 5A), metabolism dominated, followed by genetic information processing, cellular processes, and environmental information processing. At Level 2 (Figure 5B), gut microbes of all three fish groups showed significant enrichment in metabolic subpathways (e.g., global/overview maps, carbohydrate, and amino acid metabolism). At Level 3 (Figure 5C), metabolic pathways were most abundant in all three species, followed by biosynthesis of secondary metabolites. Specifically, TY had antibiotic biosynthesis and carbon metabolism; SB featured fructose/mannose metabolism, oxidative phosphorylation, and antibiotic biosynthesis; DM was enriched in oxidative phosphorylation, antibiotic biosynthesis, and carbon metabolism.

LEfSe analysis (Figures 5D,E) of KEGG metabolic pathways showed only DM and SB had statistically different biomarkers. DM had significant abundance differences in oxidative phosphorylation (Level 3, under energy metabolism, Level 2). SB showed significant abundance differences in metabolic pathways (Level 3, under global and overview maps, Level 2) and fructose/mannose metabolism (Level 3, under carbohydrate metabolism, Level 2).

PCoA (Figure 5F) on sample distance matrices revealed a significant spatial separation between SB and TY, indicating substantial KEGG functional gene composition differences. Samples within each group were close, showing small intra-group differences. Axis 1 and 2 contributed 59.76 and 15.27%, respectively.

UPGMA hierarchical clustering (Figure 5G) showed samples in each group clustered closely, indicating functional structure similarity. DM and SB had higher functional structure similarity based on branch length.

#### 3.3.2 Analysis of CAZY composition

**Figure 6:**
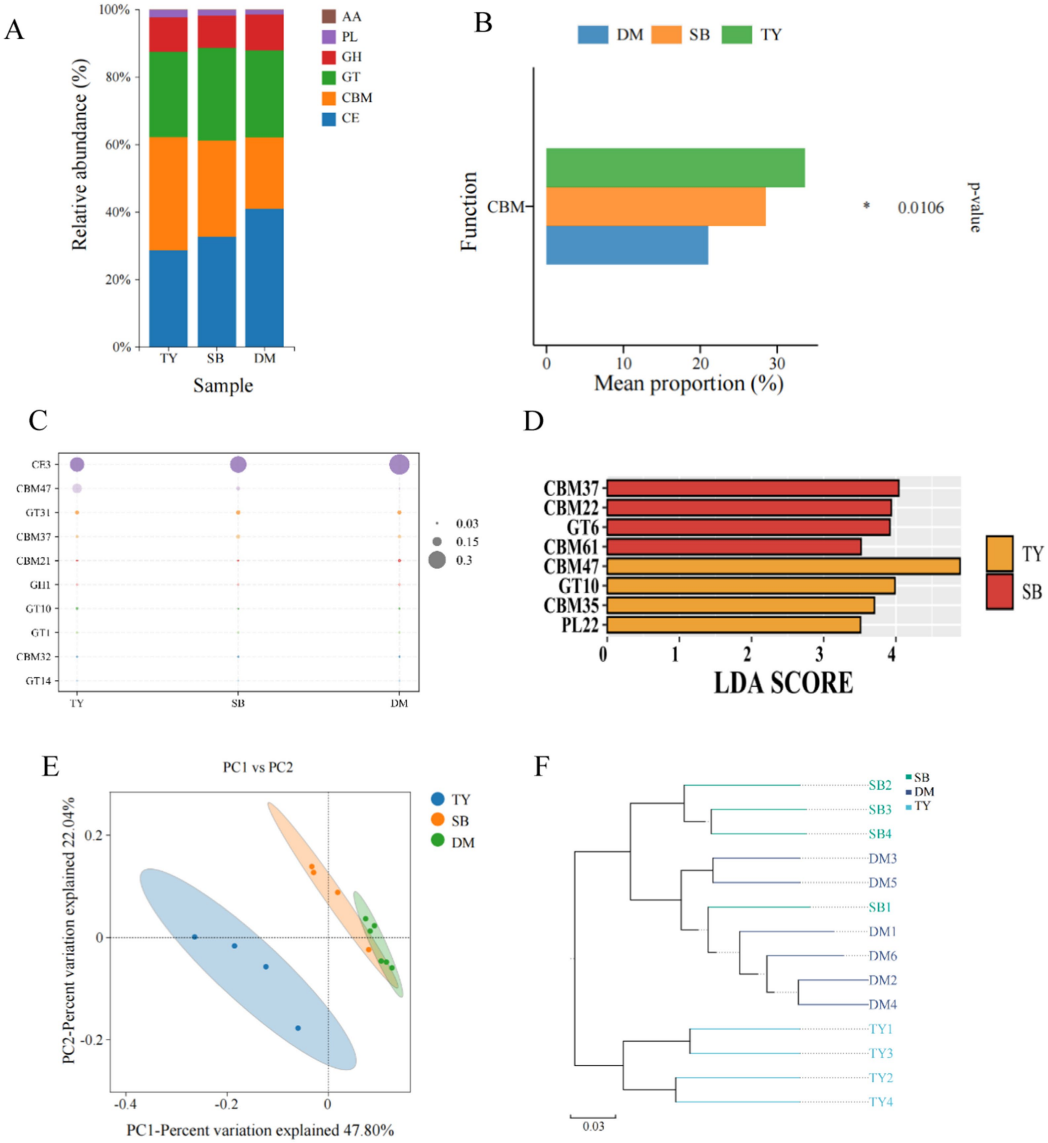
CAZy functional diversity analysis diagram. **(A)** Bar chart of CAZy functional classification abundance. X-axis: group name; Y-axis: proportion of CAZy functional categories per group. **(B)** Differential functional gene bar chart of CAZy functional classification. X-axis: average scale; Y-axis: function name. *p*-values on the far right (^*^p < 0.05). **(C)** Carbohydrate enzyme content map at CAZy family level. X-axis: sample name; Y-axis: different enzymes. Larger circle = higher relative content. **(D)** LDA value distribution histogram at CAZy family level. **(E)** PCoA of functional gene abundance via bray_curtis at CAZy family level. Dots: samples (colors = groups). X/Y-axis: principal characteristic values (percentage influence). **(F)** UPGMA clustering of functional genes via bray_curtis at CAZy family level. Colors distinguish groups; branch length: species composition similarity. DM, *Diptychus maculatus*; SB, *Schizothorax biddulphi*; TY, *Triplophysa yarkandensis*.

According to CAZy functional classification annotation (Figure 6A), TY had the highest CBM abundance, while DM and SB showed the highest CE abundance. GH, PL, and AA contents were relatively low in all three groups. The bar chart of differentially functional genes (Figure 6B) showed significant CBM differences in among the three fish species. At the CAZY Family level (Figure 6C), all three groups (TY, SB, DM) had the highest CE3 abundance, and TY’s CBM47 abundance was notably higher than that of SB and DM. LEfSe analysis (Figure 6D) revealed significant biomarkers only in TY and SB: CBM37, CBM22, GT6, and CBM61 in SB; CBM47, GT10, CBM35, and PL22 in TY. PCoA (Figure 6E) showed a significant spatial separation trend between TY and the other two species, with samples of each group being close and DM demonstrating greater consistency. Axes 1 and 2 contributed to 47.80 and 22.04%, respectively. UPGMA hierarchical clustering (Figure 6F) indicated the functional structure similarity of each group, with DM and SB showing a higher degree of similarity based on branch length.

## 4 Discussion

### 4.1 Structural characteristics of gut microorganisms of three native fish species at the phylum level

The gut microbiota is essential for maintaining aquatic animal health, influencing host development, digestion, immunity, and disease resistance (Burr et al., 2005; Yang et al., 2023). The common dominant microbial phyla of the three native fish species were Proteobacteria and Firmicutes. Although environmental, dietary, physiological, and evolutionary factors are known to significantly impact the gut microbiome in fish, multiple studies have reported the dominance of Proteobacteria (Tyagi et al., 2019; Johny et al., 2022; Hou et al., 2025), which aligns well with our findings. Liu et al. analyzed the gut microbiome of eight fish species representing four trophic levels (herbivorous, carnivorous, omnivorous, and filter-feeding) and found that Proteobacteria was a prevalent phylum among all groups (32.8–45.5%). Slightly differing from our results, this study also reported a significant abundance of Firmicutes (21.1–27.1%) and Bacteroidetes (0.9–8.26%) in the gut microbiome of fish (Liu et al., 2016).

Literature has presented Firmicutes and Bacteroidetes as the main components of the gut microbiota in land animals, with relatively lower prevalence in fish intestines (Li T. et al., 2015; Liu et al., 2016; Tyagi and Singh, 2017). Overall, these studies reveal the dynamic host specificity of gut microbiota taxonomic composition in fish. Exploring microbial composition and metabolic pathways (discussed later) is crucial for understanding their impacts on host physiology. Among the identified Proteobacteria phylum in all three native fish species, the γ-Proteobacteria class was most abundant, similar to findings in herbivorous species like grass carp (Han et al., 2010) and omnivorous carp (Meng et al., 2018). Most lignin-degrading bacteria belong to the γ- and α-Proteobacteria classes, which actively degrade lignin and disrupt plant cell walls to aid cellulose/hemicellulose digestion (Bugg et al., 2011; Ausec et al., 2012). Chen et al. (2014) found that plant debris comprised a high proportion of TY’s gut contents in March. Li (2023) reported DM as omnivorous, while Zhao H. et al. (2022) indicated that SB is omnivorous-carnivorous with seasonal feeding shifts. During April sampling, the Kizil River’s low water temperature likely reduced zooplankton and benthic food availability, forcing the fish to rely on phytoplankton and plant debris. This dietary shift from omnivory to herbivory may explain the colonization of Proteobacteria to assist plant polymer digestion. Firmicutes, the second most abundant in SB and TY, play a role in hydrolyzing proteins and carbohydrates (Bahar-Tokman et al., 2022), which significantly contributes to nutrient absorption and metabolism in the host. It has been reported as a dominant gut microbe in several fish studies, including rainbow trout (*Oncorhynchus mykiss*) (Kim et al., 2007), grass carp (*Ctenopharyngodon idellus*) (Jiang et al., 2011), and *Silurus lanzhouensis* (Ruan et al., 2023). Among herbivorous, omnivorous, and carnivorous fish, Firmicutes dominance tends to decrease sequentially (Menke et al., 2014; Xu, 2022). Among the gut microbiota of the three native fish species studied, *Firmicutes* exhibited relatively high diversity and abundance. However, microbial composition does not fully conform to dietary rules, indicating their complex diet may shift toward omnivorous and herbivorous. Despite species differences among the three types of fish, they rely on similar microbial groups for nutrient acquisition and basal metabolism, reflecting the core supporting role of gut microorganisms in the survival of fish. The Firmicutes-to-Bacteroidetes (F/B) ratio is widely linked to gut environmental stability (Stojanov et al., 2020). Higher ratios correlate with better fish health (Li et al., 2024) and enhanced nutrient utilization (Liu X. et al., 2023). DM and SB showed no significant F/B ratio differences but had higher values than TY, indicating stronger nutrient handling and gut stability in DM/SB compared to TY. In SB, Proteobacteria and Firmicutes as dominant biomarkers align with its omnivorous-carnivorous diet (Zhao H. et al., 2022). *Actinomycetes*, Gram-positive bacteria, produce bioactive secondary metabolites that reduce pathogen virulence (Barka et al., 2016). Studies show higher *Actinomycetes* abundance correlates with better gut health (Liu X. et al., 2023). They dominate the gut microbiota of many freshwater fish, including rainbow trout (*Oncorhynchus mykiss*) (Hai et al., 2023), *Silurus lanzhouensis* (Ruan et al., 2023), *Micropterus salmoides* (Liu X. et al., 2023), and *Squaliobarbus curriculus* (Zhao et al., 2023). *Actinomycetes* has been found to be a dominant gut microorganism. As a phylum-level biomarker for DM, Actinobacteria may support winter survival under cold and starvation stress. Mucoromycota, a phylum reclassified from Zygomycetes by Spatafora et al. (2016), remains relatively unexplored. These terrestrial fungi grow rapidly and are widely distributed in decaying plant matter and soil. Mucoromycota is identified as a biomarker in TY, and in DM it is also higher than that of SB. This suggests that DM and TY had distinct basal feeding habits during the study period, and *Mucoromycota* was ingested into the intestine along with plant debris during the study period.

### 4.2 Structural characteristics of gut microorganisms of three native fish species at the genus level

In this study, among the 20 most dominant genera in the gut microbiome of fish, eight belong to *Proteobacteria*. These genera collectively account for 15.6, 32.99, and 30.86% of the total genus diversity in the gut microbiome of TY, SB, and DM, respectively. *Acinetobacter* and *Pseudomonas* are common dominant microbial genera in all three indigenous fish species. Both are Gram-negative, aerobic, and opportunistic pathogenic bacteria found in the gut of aquatic animals (Jung and Park, 2015). Such potential pathogenic bacteria naturally colonize the microbiome of fish, but their pathogenicity is observed only when the host is under stress (Tao et al., 2022). These bacteria have been reported in the intestines of farmed fish such as grass carp (*Ctenopharyngodon idellus*) (Chen et al., 2018) and *Tinca tinca* (L) (Chen and Feng, 2020), where they cause bacterial sepsis, enteritis, etc. The abundance of these bacteria in native fish species during the overwintering period might be linked to relatively poor immunity. This could be due to decreased food intake during overwintering and the depletion of nutrients such as protein and fat, which significantly reduces their immunity. *Acinetobacter* has been recognized as an indicator of gut health in many farmed fish species, including largemouth bass, *Gifu tilapia*, Atlantic salmon, and bastard halibut (Qiang, 2023). The content of *Acinetobacter* in DM and SB was found to be higher than in TY, suggesting that DM and SB may be more susceptible to diseases caused by this bacterium. Therefore, it is necessary to strengthen monitoring of bacterial pathogens such as *Acinetobacter* in the aquaculture water environment and during the management of DM and SB, to prevent and control related disease outbreaks. The biomarker of SB, *Aeromonas*, is a Gram-negative aerobic or facultative anaerobic bacterium known for its cellulose-decomposing ability. Previous studies have shown that *Aeromonas caviae* produces cellulase, helping the host break down ingested cellulose and hemicellulose (Sharma et al., 2022). The role of *Aeromonas* in assisting SB with cellulose breakdown aligns with these findings. Another biomarker of SB is Bacillus, which can produce short-chain fatty acids, promote the adhesion of healthy probiotics to the gut mucosa, produce antibacterial substances, and modulate immunity to maintain gut barrier function and improve disease resistance during fish development (Fan et al., 2023b). This indicates a healthier gut state in SB, consistent with the F/B ratio results mentioned earlier in the text. The genus-level biomarker for DM is *Micromonospora*, an actinomycete with strong chitinase production and cellulose-degrading activity. In previous studies, *Micromonospora* has mostly been identified as a dominant gut microorganism in insects but is rarely reported in fish (Qiang, 2023). Some of its strains also produce xylanase and cellulase, which can effectively aid DM in digesting cellulose, hemicellulose, and crustacean chitin.

The biomarker of TY is *Alcanivorax*, a bacterium that often dominates in crude oil-contaminated temperate seawater and has been identified as a key heterotrophic microorganism in alkane degradation, utilizing limited carbon sources, including alkanes. It serves as an indicator of alkane contamination in marine environments (Koike et al., 2023). The abundance of *Alcanivorax* in TY indicates possible crude oil contamination in the Kizil River and suggests that TY is more sensitive to environmental pollution. *Vibrio* is another biomarker of TY, existing as a symbiont in the gastrointestinal tract of fish, generating hydrolases to decompose feed components. It is reported that *Vibrio* can produce amylase, cellulase, chitinase, and lipase (Johny et al., 2022). *Vibrio* as a biomarker of TY aligns with previous speculations about dietary influence. *Epsilonretrovirus* is also a biomarker of TY, although it appeared more abundant in the gut tract of DM than in SB. This might be attributed to the consumption of substantial substances and energy during overwintering, leading to reduced immunity. TY appears more sensitive to environmental stress, while SB may be more adaptable to environmental changes. Finally, both *Thalassococcus* and *Kangiella*, with significant abundance differences in TY, are associated with marine environments, indicating TY’s ability to adapt to a wide salinity range, consistent with its migratory habits.

### 4.3 Structural characteristics of gut microorganisms of three native fish species at the species level

The common dominant microbial species of all three indigenous fish species were *A. baumannii*, *Pseudomonas stutzeri*, and *Gammaproteobacteria bacterium*, all belonging to the γ-Proteobacteria class. Although literature supports the role of *A. baumannii* in human clinical infections (e.g., pneumonia, sepsis) as an opportunistic multidrug-resistant pathogen, its role as a fish pathogen has been rarely reported (Xia et al., 2008). Some studies note that *A. baumannii* is not commonly associated with fish microbiota (Gheorghe-Barbu et al., 2024), but Xia et al. (2008) and Xu et al. (2023) identified it as a pathogen in catfish (*Ictalurus punctatus*) and Atlantic salmon (*Salmo salar*). In salmon, this conditional pathogen increased significantly under low dissolved oxygen, accompanied by a strong immune response. The colonization of *A. baumannii* in freshwater fish is concentration-dependent, with low natural water concentrations correlating with limited colonization (Dekić et al., 2018). Its dominance in these Xinjiang fish may reflect either hypoxic stress triggering immune responses or elevated *A. baumannii* levels in the Kizil River, highlighting the need for preventive management (e.g., non-antibiotic strategies) to reduce infection risks. Fish carrying *A. baumannii* pose a potential zoonotic transmission risk through handling or consumption, though its virulence in mammals requires further study. *Pseudomonas stutzeri*, capable of removing nitrogen via assimilation, nitrification, and denitrification, shows promise for wastewater treatment, though its aquaculture applications remain underexplored (Fu et al., 2023). Its abundance in these fish suggests elevated nitrogen levels in the Kizil River during sampling, possibly from pollution or algal decomposition. *Gammaproteobacteria bacterium* has over 110 functional roles, mostly linked to vital activities, and has been isolated from mosquitoes (Zhao T. et al., 2022). *Alcanivorax* sp. *MTEO17T*, a Gram-negative, rod-shaped, non-motile, strictly aerobic strain, was isolated from 1,000 m deep seawater (Liu et al., 2019). Montes et al. (2008) isolated two strains of Gram-negative, cold-adapted, moderately halophilic, aerobic bacteria belonging to the same genetic species from the marine sediments of the South Shetland Islands in Antarctica, representing a new species, *Marinaprus guineae* sp. nov. *Aeromonas P2973* was isolated from a freshwater brook in the Czech Republic cave and, based on a polyphasic approach, was proposed as a novel species, *Aeromonas cavernicola* sp. nov., with strain CCM7641^T^ (DSM24474^T^, CECT7862^T^) as the type strain, within the genus Aeromonas (Martínez-Murcia et al., 2013). *Micromonospora* sp. *RP3T*, as a potential new member of the *Micromonospora (Actinobacteria)*, may contribute to the production of active metabolites, environmental remediation or host interactions. The relevant studies on the biomarkers mentioned above are limited in their thorough discussion and analysis. The host and the gut microbiota are interdependent, maintaining a dynamic balance and a reciprocal relationship (Duncan et al., 2023). Given that the habitat of gut microorganisms is significantly controlled by the host’s physiology, only bacterial species adapted to this environment can thrive in the intestine (Sadeghi et al., 2023). The host-related microbiota, especially the bacterial communities existing within and on the body surface of the host (hereinafter referred to as the microbiome), influence a wide range of host immunity, evolution, and ecological processes. According to systemic symbiosis theory, host-microbiome phylogenies are linked, predicting closer microbial similarity in phylogenetically related hosts (Brooks et al., 2016). Here, alpha diversity analysis showed higher species diversity/homogeneity in TY than other groups, despite similar richness. DM and TY shared more dietary traits than SB, aligning with Johny et al. (2022). While diet influences microbiota, species-specific effects persisted after dietary adjustment, indicating host phylogeny as a primary driver. Beta diversity analysis revealed distinct microbiota structures among species. Cluster tree branch lengths showed greater DM/SB similarity (both Cyprinidae) than TY (Cobitidae), supporting host species as a key microbiota determinant, consistent with Sadeghi et al. (2023). The host-specific microbiome is ubiquitous in nature and also exists in many fish hosts (Doane et al., 2020; Johny et al., 2022; Minich et al., 2022). Although some bacterial lineages may still be co-diverse with their hosts, it should be noted that systemic symbiosis does not conclusively explain host-microbiome adaptive coevolution. Current evidence of systemic symbiosis in non-mammalian vertebrates is inconsistent (Johny et al., 2022). For instance, some fish studies have shown evidence of systemic symbiosis (Doane et al., 2020; Minich et al., 2022), while other studies reported varied results and weak evidence (Riiser et al., 2020). Further research is needed to clarify host-microbe coevolutionary dynamics.

### 4.4 KEGG functional characteristics of gut microorganisms in three native fish species

Among the three types of fish, KEGG level 1 classification revealed the highest proportion of Metabolism, followed by genetic information processing, cellular processes, and environmental information processing. These findings are consistent with the results reported by Ma et al. (2019) on the gut microbiota of rats under hypoxic exposure. During sampling in this study, the three native fish species had just emerged from the wintering period. The temperature and oxygen content of the snowmelt water collected from the Kizil River were relatively low. The sampling sites were located at an average altitude of approximately 3,200 meters. The high-altitude and hypoxic environment may have caused genomic stress and damage to the host’s gut microbiota, possibly explaining the enrichment of genetic information processing pathways. The greatest challenge for the three native fish species during overwintering in such harsh conditions was survival. To cope with complex external environmental stresses, the species rely on gut microorganisms to assist in digesting food and replenishing energy. The microbiota also utilize energy and substances from the host’s ingested food to ensure their survival, proliferation, and mutualistic functions, such as potentially repairing genetic damage caused by hypoxia. Studies have demonstrated significant differences in microbiome-predicted functions across animal taxa, influencing processes like carbohydrate, lipid, and amino acid metabolism (Fan et al., 2023b). However, the specific mechanisms by which the microbiome influences host physiology remain unclear. KEGG pathway analysis of gut microbiota metabolic functions showed carbohydrate metabolism, amino acid metabolism, nucleotide metabolism, and energy metabolism as dominant categories. This aligns well with research on *Hexagrammos otakii* (Fan et al., 2023a) and red swamp crayfish (*Procambarus clarkii*) (Liu S. et al., 2023), suggesting gut microbiota meet growth requirements via specific pathways. Microbes involved in these functions, such as Firmicutes in all three fish and SB’s biomarker *Bacillus*, are relatively abundant. These microbes ferment carbohydrates and proteins in the intestine, aiding nutrient and energy acquisition from the diet. Functional predictions further indicated cofactor, vitamin, amino acid, and nucleotide metabolism pathways. Among these, abundant energy and amino acids play critical roles as mediators in biochemical conversions and carbohydrate metabolism, which are fundamental to gut microbial activity and host survival (Liu S. et al., 2023).

Carbohydrates are an important source of energy for fish and microbial cells. Plant components contain a large amount of carbohydrates, but compared to mammals, fish are generally regarded as poor users of carbohydrates. Typically, omnivorous and herbivorous fish can utilize 15–25% of carbohydrates, while carnivorous fish have a lower carbohydrate utilization efficiency (Zhao L. et al., 2022). Nondigestible carbohydrates are fermented by gut microbiota in the colon, yielding energy for microbial growth and promoting gut health (Fan et al., 2023a). Microbial fermentation of carbohydrates under anaerobic conditions results in the production of short-chain fatty acids (SCFAs) such as butyrate, propionate, and acetate, which can be utilized by the host. Ibrahim and Anishetty (2012) found that propionic acid is an important metabolite in high-altitude adaptation, which indirectly supports the responses of three native fish species to high-altitude habitats. The main enrichment pathways in carbohydrate metabolism were fructose and mannose metabolism, which was significantly higher in SB (as a biomarker) than in DM and TY. This pathway converts fructose and mannose into glucose-6-phosphate (G6P) or fructose 6-phosphate (F6P), which is connected to the glycolysis/gluconeogenesis pathway to generate cellular energy or synthesize precursors (such as glycogen and nucleotides). This may be interpreted as follows: (1) It is closely related to the diet rich in plant polysaccharides consumed by SB during this period, reflecting the functional adaptation of gut microbiota to the host’s diet. (2) The efficient operation of this pathway may enhance the energy utilization efficiency of the host. Meanwhile, the generated SCFAs can regulate gut immunity, forming a positive cycle of “microbiota-metabolism-immunity.” (3) In the gut bacteria of omnivorous fish, Bacteroidetes and Firmicutes often carry more carbohydrate active enzymes (CAZy), which is consistent with the following results. (4) The findings provide a basis for optimizing carbohydrate-based feed in aquaculture, such as designing fructose/mannose formulas for omnivorous fish to promote metabolic synergy between gut microorganisms and the host. In this study, the main enrichment pathway related to energy metabolism is oxidative phosphorylation. As a biomarker for DM, oxidative phosphorylation is a process that utilizes energy generated from the oxidation and decomposition of organic substances, such as carbohydrates, fats, and proteins, for ATP synthesis through the electron transport chain. It is the most efficient way for aerobic organisms to generate energy.

It may indicate that the energy metabolism of the three native fish species in Xinjiang is highly dependent on the oxidation and decomposition of organic matter. Given their carnivorous or omnivorous diet, organic matter rich in food can be efficiently decomposed through oxidative phosphorylation, allowing ATP to be rapidly produced to meet high energy demands, such as active predatory behavior after the overwintering period. Therefore, the predatory behavior of DM may be more pronounced. According to Ibrahim and Anishetty (2012), a greater ability to obtain energy from the diet. The F/B ratio observed in this study aligns well with the energy metabolism findings. During the April sampling, the Kizil River’s water temperature was relatively low, and zooplankton, benthic animals, and other food organisms were scarce. These conditions decreased fish activity, reducing their ability to hunt. Fish in long-term, highly competitive energy environments may evolve efficient oxidative phosphorylation pathways to utilize limited organic resources. The abundance of the oxidative phosphorylation pathway in TY was the lowest, likely due to its distinct bottom-dwelling feeding habits and minimal dietary competition with the other two species. Additionally, the gut microbiota plays an important role in fish metabolism. The fish gut tract is rich in microorganisms involved in oxidative phosphorylation, which can enhance this metabolic pathway. However, in the study by Fan et al. (2023b) on *Hexagrammos otakii*, the main enrichment pathway of energy metabolism was carbon fixation, contrary to our findings. Carbon fixation is the process of converting inorganic carbon (e.g., CO₂) into organic carbon (e.g., sugars), typically through photosynthesis (e.g., photosynthetic bacteria) or chemosynthesis (e.g., certain autotrophic bacteria). This is consistent with the presence of the taxonomic biomarker *Photobacterium* in the juvenile stage of *H. otakii*. The consistency between microbial composition and functional pathways indicates that the functional potential of the microbiota may increase or decrease with changes in microbiota diversity.

Beta diversity analysis revealed significant differences in KEGG functional pathways between TY and SB gut microbiota. Based on cluster tree branch lengths, DM and SB showed higher similarity, with DM sharing overlapping dietary and ecological niches with the other two species. These findings suggested that the host species is primarily the main factor driving the functional differences, although diet might also impact these differences. The results are generally consistent with those of the species composition of gut microbiota, indicating a strong correlation between the functional composition of the metagenome and the taxonomic composition. A similar finding was also reflected in a study by Fan et al. (2023a).

Currently, the results of our relevant research are based solely on sequencing data and predictions of functional pathways, with relatively few verification data available. Further experimental validation is necessary to elucidate the underlying mechanisms influencing these microbial functions.

### 4.5 CAZY functional characteristics of gut microorganisms in three native fish species

Although fish have the enzymes needed for digestion, the gut microbiota also plays an important role in breaking down complex carbohydrates (Johny et al., 2022). CAZY annotation results showed that TY, SB, and DM all had the highest abundance in CE3. By comparing KEGG and CAZY annotations, CE3 was found to be involved in fructose and mannose metabolism, with consistent abundance patterns. CE3 genes encode CAZymes responsible for degrading xylan and other related substances, consistent with *Actinomycetes* (a DM biomarker) producing xylanase and cellulase. This aligns with the species’ dietary behaviors: during sampling, all three shifted to omnivory with a herbivorous bias, although DM’s shift was more pronounced, and TY’s was less so.

Within the functional classification of CAZy, the abundance of the carbohydrate-binding functional domain (CBM) biomarker in TY is higher than that in DM and SB. The non-catalytic CBM family does not have independent enzymatic activity but serves as a substrate-binding module, typically functioning in conjunction with the other five families (Liang, 2023). It facilitates the enzymatic degradation of carbohydrates by attaching to polysaccharide substrates. CBM, whether as single or multiple domains, is approximately 30 to 200 amino acids in length, connected to either the C or N terminal of the catalytic domain. Some believe that the binding characteristics of CBM improve the catalytic function of the enzyme by increasing the proximity between the enzyme and its target substrate, thereby disrupting the crystallization of insoluble substrates (Teo et al., 2019). Therefore, CBM is considered to enhance enzymatic hydrolysis and plays an important role in many biotechnology applications, particularly in improving the degradation efficiency of polysaccharides (Sidar et al., 2020). It is speculated that CBM enhances the functions of GT (glycosyltransferases), GH (glycoside hydrolases), and PL (polysaccharide lyases) in TY and SB. LEfSe difference analysis at the CAZy family level revealed statistically significant biomarker differences between TY and SB. The CBM abundance in DM was found to be the lowest, which is consistent with the previous result indicating that CBM enhances enzymes of other families. In SB, GT6 showed significant abundance differences. The significant abundance differences in TY were observed in GT10 and PL22. Coelho et al. (2018) reported that GT6 was more abundant in the gut microbiome of dogs on high-protein/low-carbohydrate (HPLC) feed. The enrichment of GT6 in SB suggests that despite the dietary shift toward a more herbivorous diet, it remained predominantly omnivorous with a preference for carnivory. Although the function of this ubiquitous glycosyltransferase (GT) enzyme in bacteria is still unclear, it generally catalyzes the formation of many different types of glycoproteins, which play important roles in intercellular communication, recognition, and the utilization or recycling of carbohydrates (Coelho et al., 2018).

Pectate lyases (PLs) are enzymes involved in the degradation of plant cell walls by cleaving pectin using a β-elimination mechanism, specific for acidic polysaccharides. These enzymes can be produced by gut bacteria that digest plant material in the digestive tracts of their hosts (Hugouvieux-Cotte-Pattat et al., 2014). The degradation products of pectin may serve as signaling molecules influencing plant-bacterial interactions, reflecting the symbiosis between the host and gut microbiota. The high abundance of PL22 in TY indicates a greater intake of algae and other plant substances compared to SB and DM, supporting the previous inference of relatively small changes in the diet of TY during the overwintering period.

Beta diversity analysis showed that TY’s CAZY functional profiles were distinct from those of DM and SB. DM and SB exhibited higher similarity, likely due to similar basal feeding conditions; however, host species remained the primary driver of microbiome functional differences, aligning with the study by Doane et al. (2020) on microbiome host specificity. Our CAZY annotations and microbiota composition showed strong consistency, linking metagenomic function to taxonomic composition. Currently, the results rely on sequencing and predictions, with limited experimental validation, necessitating further mechanistic research.

## 5 Conclusion

This study employs shotgun metagenomics to comprehensively compare gut microbiome diversity, metabolic pathways, and CAZY functions among three Xinjiang native fish species, revealing that host species are the primary drivers of taxonomic and functional differences in gut microbiota. The dominance of metabolic functions (KEGG pathway analysis) highlights the critical role of gut microbes in energy and nutrient extraction from diets and their influence on host physiological development via metabolites as signaling molecules. These findings provide a scientific basis for understanding host-microbiome interactions, supporting the ecological conservation of native fish, and optimizing aquaculture practices by targeting microbial functions.

Current results rely solely on sequencing data and functional pathway predictions, lacking experimental validation of microbial mechanisms. The taxonomic and functional correlations observed require further mechanistic studies, such as *in vitro* or *in vivo* assays, to confirm metabolic pathway activities. Additionally, while *Acinetobacter baumannii* was identified in all three species, its virulence potential in mammals and specific mechanisms of host-pathogen interaction remain uncharacterized, and the ecological significance of its dominance in fish gut microbiomes is poorly understood.

To address these gaps, future studies should validate microbiome-predicted functions through experimental methods (e.g., microbial culture, gnotobiotic models), investigate *A. baumannii* virulence traits and transmission risks (including antibiotic resistance and zoonotic potential), integrate long-term environmental monitoring to assess water quality impacts on gut microbiome stability and pathogen proliferation, and explore probiotics and dietary interventions to modulate gut microbiota for enhanced host health in aquaculture, thereby increasing the translational value of metagenomic findings and supporting the sustainable management of Xinjiang’s native fish resources.

## Data Availability

All the raw data have been deposited in the NCBI Sequence Read Archive database (SRA accession number is PRJNA1254096).
